# Both trait-neutrality and filtering effects are validated by the vegetation patterns detected in the functional recovery of sand grasslands

**DOI:** 10.1038/s41598-018-32078-x

**Published:** 2018-09-12

**Authors:** P. Török, G. Matus, E. Tóth, M. Papp, A. Kelemen, J. Sonkoly, B. Tóthmérész

**Affiliations:** 1 MTA-DE Lendület Functional and Restoration Ecology Research Group, Egyetem tér 1, H-4032 Debrecen, Hungary; 2 University of Debrecen, Department of Ecology, Egyetem tér 1, H-4032 Debrecen, Hungary; 3 University of Debrecen, Department of Botany, Egyetem tér 1, H-4032 Debrecen, Hungary; 4 MTA Postdoctoral Research Program, MTA TKI, Nádor utca 7, Budapest, H-1051 Hungary; 5 MTA-DE Biodiversity and Ecosystem Services Research Group, Egyetem tér 1, H-4032 Debrecen, Hungary

## Abstract

Neutral theory of species assembly means that species assembly is governed by stochastic dispersal processes and fluctuations in established populations. An alternative theory suggests that assembly is strongly determined by functional trait filtering governed by abiotic and biotic filtering selecting species from the local species pool. To test these assumptions, in the current paper we analysed vegetation changes in the first 12 years of succession after heavy goose grazing on acidic sand. With trait-based analyses using permanent plots we addressed the following hypotheses: (i) High fluctuations in the trait values are typical in the first years; later a temporally divergent change in the trait patterns of sites with different vertical position became characteristic. (ii) In the functional diversity of regenerative and vegetative traits we expected different temporal patterns. We confirmed the first hypothesis, as in the first few years most traits displayed high fluctuations with no clear patterns. Our findings weakly supported the second hypothesis; while there were distinct patterns detected in the functional richness of traits, functional divergence and evenness displayed no clear distinctive patterns. We can conclude that both trait neutrality and filtering effects operate in the vegetation changes of the first period of secondary succession.

## Introduction

Grassland ecosystems are suffering worldwide from area loss and changing levels of human pressure mostly in form of altered management ranging from intensification to underuse and abandonment^1^. To mitigate the negative effects of human pressure and to facilitate the recovery of grasslands, it is vital to understand dynamic processes that govern species assembly and processes related to the changes in biodiversity. One of the most exciting species assembly processes is vegetation succession. The early studies dealt with the description and analysis of general changes in vegetation patterns, but later the link between pattern and processes has become more important^2^. Driven by socio-economic and environmental changes, there is an increasing trend of land abandonment worldwide, which underlines the necessity to understand processes that govern vegetation succession in abandoned agricultural areas^3^.

There are several contrasting views of species assembly and the course of succession. Neutral theory of species assembly (in sense of Hubbel^4^) suggests that species assembly is rather a stochastic process and it is governed by dispersal processes (e.g. priority effects and differences in species arrival and/or seed banks) and stochastic fluctuations in established populations. This view is deeply rooted in the view of Gleason^5^ and in the initial floristic composition theory of Egler^6^ (revisited by Wilson *et al*.^7^). Another approach suggests that community assembly is strongly determined by functional trait filtering governed by more or less definite interaction of abiotic and biotic filtering processes selecting species from the available local species pool. This means that the vegetation development processes are rather predictable based on the functional trait composition of the community. This view is strongly linked to the classical deterministic successional theory of Clements^8^, improved by several authors including Drury & Nisbet^9^ or Conell & Slatyer^10^. Field evidence suggests that due to differences in local species pools, dispersal processes, site characteristics, and climate, multiple pathways of succession occur. This implies that neutrality and filtering both operate in communities; however, their spatial and temporal patterns may differ within and between different species assemblies^11^. Former research suggests that early vegetation development is mainly driven by stochastic fluctuations and dispersal interacting with local abiotic conditions, while biotic habitat filtering (e.g. by facilitation or competition) becomes an important driver later on^12^.

The trait-based approach may provide a powerful tool to understand the mechanisms of temporal changes, species assembly and biotic interactions^13,14^. Using traits instead of studying changes in taxonomic composition also enables us to detect general trends and processes directing vegetation changes in similar habitats but with different flora^15^. Studying changes in the trait composition was increasingly considered in studies of vegetation succession in the last decade, but most interest was directed to forest succession so far (but see Purschke *et al*.^16^ or Douma *et al*.^17^).

Most studies report trait-based analyses of vegetation development comparing the vegetation along a chronosequence^17–20^. Although studies of chronosequences are useful in detecting general patterns of vegetation change^21^, the most interesting early-phase filtering and assembly processes remained hidden because of broad age categories used in most studies. In the current paper we analysed vegetation patterns during the first 12 years of secondary succession after heavy goose grazing in an acidic sand area. We used a permanent plot setup and trait-based analyses to address the following hypotheses: (i) Former research suggests that early vegetation development is characterised by stochastic processes and the interaction of abiotic and biotic filtering become increasingly important driver of vegetation changes in the later period^12,22^. Thus, we expected high fluctuations in the trait values in the first years; and later a temporally divergent change in trait patterns of sites with different vertical position. (ii) It was pointed out that regenerative traits like seed bank formation and dispersal type are in general more crucial for the vegetation development in the first period of succession, while vegetative traits related to competition and establishment become more important later^19,23^. Thus, we expected different temporal patterns in the functional diversity of regenerative and vegetative traits.

## Methods

### Study site and vegetation sampling

Our sample sites were located at the Martinka Pasture nature reserve situated 15 km to the east from the city of Debrecen, East Hungary (a 2.8 km^2^ large area situated N 47°34′00″–35′20″, E 21°46′30″–48′40″). The climate of the nature reserve is moderately continental with a mean annual precipitation of 600 mm and a mean annual temperature of 10 °C, but large fluctuations in mean temperatures and precipitation are rather typical^24^. In the studied time period of 12 years, 1998, 1999 and 2002 were those with high precipitation (i.e. with at least 25% above the average), whereas the dry years (i.e. with at least 25% below the average) were 1992, 1993 and 2000. The study area is covered with 3–10 m high dunes formed from acidic sand, separated with flat dune slacks and channels. The vegetation is characterised by sand grassland communities at the dune tops and slopes and with various wet meadows and marshes in the dune slacks.

**Figure 1:**
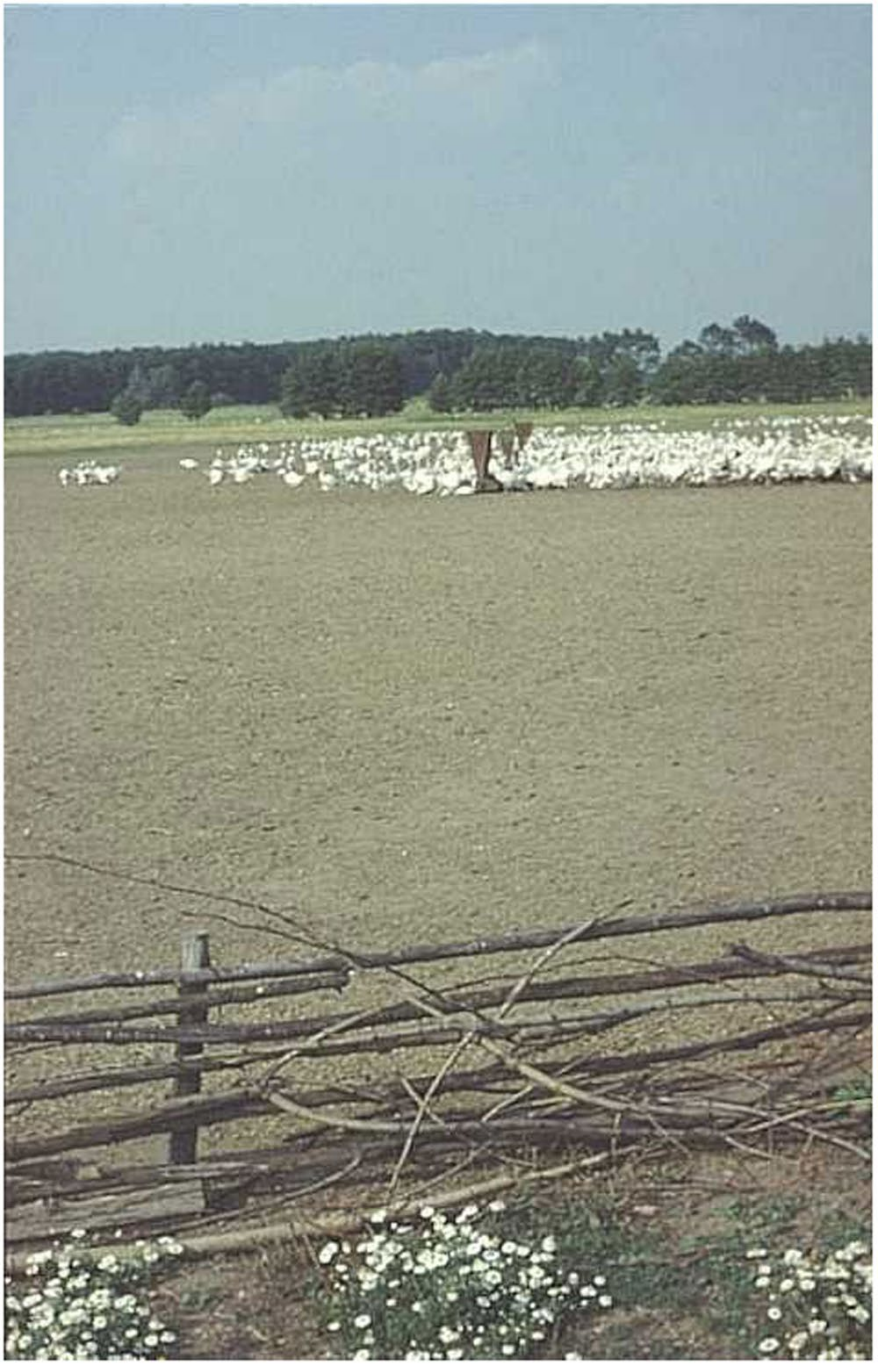
A typical goose farm in 1989 established in a former sand grassland stand in the study area. (photo by G. Matus).

The area was traditionally used for cattle and sheep grazing, but in the late '80s large areas were fenced and tens of thousands of domestic geese were kept with supplementary feeding until the denudation of the original vegetation^24^. After the almost complete elimination of the vegetation the fences were translocated to another place covered with grassland vegetation (Fig. 1). This type of management resulted in a large-scale degradation of the sandy grassland areas, but also provided excellent objects for studying secondary grassland succession.

Four sites located at former goose farms with different elevation were selected for the study. All farms were abandoned in 1990 and vegetation recording started in the following year, 1991. Two sites were situated at 2.1–2.9 m (measured from the dune slack), near to the dune top (in the followings high positioned sites U1 and U2) and two sites at the lower part of the dune slope, at 1.5 m measured from the dune slack (in the followings low positioned sites L1 and L2). Two sites were located at the eastern (U1, L1), while two sites (U2, L2) at the western part of the nature reserve. The sites were subjected to low intensity sheep and cattle grazing and spontaneous succession during the study period. At each site, altogether five, 4-m^2^ plots were permanently marked in 1991, and the vegetation cover was recorded using a percentage scale in every year in early summer.

### Traits

Altogether 15 functional plant traits were considered in the analyses. Out of the analysed traits there were seven traits related to growth, vegetative spread and competitive ability of plants (referred to as ‘vegetative’ traits in the followings) and eight regenerative traits including traits of generative reproduction, spatial dispersal and persistence (based on the classification of Purschke *et al*.^16^).

As vegetative traits we included four leaf traits (leaf area, leaf dry weight, leaf dry matter content and specific leaf area), plant height, life form and clonal spreading ability into the analyses. Leaf area (LA), leaf dry weight (LDW), leaf dry matter content (LDMC) and specific leaf area (SLA) were obtained either (i) from the LEDA trait database^25^, (ii) from local measurements of Lhotsky *et al*.^26^ (iii) or we used own measurements obtained by using standardised measurement protocols^27^. To obtain plant height and life form data we used the reference work of the Pannonian flora^28^. For the plant height we considered the mean of the plant height extremes provided for each species. Qualitative data for plant life form were transformed to an ordinal scale based on the potential life span of each category: (1) annuals, (2) biennials, (3) hemicryptophytes and geophytes, (4) chamaephytes. For clonal spreading ability we used the CLO-PLA database^29^ and classified the species into four ordinal categories based on potential distance of lateral spreading: (1) no clonal spreading, (2) < 0.01 m/year, (3) 0.01–0.25 m/year, and (4) >0.25 m/year.

Eight traits related to generative reproduction were considered: flowering start, flowering period, the rate of wind pollination, insect pollination and self-pollination, seed weight and terminal velocity (as proxies for seed dispersal), and seed bank type. The starting month of flowering (on an ordinal scale) and flowering period (number of months of flowering) were obtained from Király^28^. For pollination type (wind pollination, insect pollination and likeliness of self-pollination) we used presence/absence data from the LEDA^25^ and BiolFlor databases^30^ and missing data of species were completed by our own evaluations of flower morphology. As proxies of spatial seed dispersal we used datasets of seed weight and terminal velocity. Seed weights were obtained from Török *et al*.^31,32^ and from other own unpublished seed weight data of the Pannonian flora, while terminal velocity scores were obtained from the LEDA traitbase^25^ or the D3 database^30^. For seed bank types we used the ordinal classification of Thompson *et al*.^33^ with three categories: (i) transient, (ii) short-term persistent and (iii) long-term persistent. We used two sources of seed bank types: first, species data were obtained from Thompson *et al*.^33^ where longevity indices (LI) were calculated based on the published records (i.e. the proportion of non-transient records in the database). These data were transformed to an ordinal scale (i.e. transient = LI ≤ 0.33, short-term persistent = 0.34 ≤ LI < 0.67, and long-term persistent = 0.67 ≤ LI). We also obtained seed persistence data from datasets of local experts who have been collecting persistent seed bank data of the Pannonian flora for more than two decades (Matus & Török unpublished data). The missing species data were completed using this latter-mentioned data source and other published seed bank records of the region^34,35^.

### Data processing and analyses

For a limited set of low cover species we could not obtain some of the trait data; thus, we omitted these species from the analyses (*Amaranthus albus*, *Crataegus monogyna*, *Hibiscus trionum*, and *Trifolium diffusum*). Horsetail species *Equisetum arvense* and *E. ramosissimum* were not considered during the analyses of pollination and dispersal traits. We calculated community-weighted means (CWMs) and single trait variance (FDVar) for each trait, and multi-trait functional richness (FRic), functional evenness (FEve), and functional divergence (FDiv)^36,37^ using all traits in exception of presence/absence traits of pollination. We also calculated the multi-trait indices separately for the vegetative and regenerative trait groups. For the calculation of all the indices, we used the FDiversity program package; we used Gower distance measure using species cover scores for weighting^38^. To analyse the effect of total cover change on each of the functional diversity metrics, we calculated Spearman rank-correlation.

Temporal and spatial trends in the datasets were analysed using two-way repeated measures GLM, where ‘site’ was included as fixed factor, ‘successional time (years)’ was included as repeated measure factor. Dependent variables were the followings: cover weighted CWMs and FDVar of all traits, FRic, FEve, and FDiv overall and considering the group of vegetative and regeneration traits separately. For significant site-dependent effects validated by the two-way repeated measure GLMs, we used one-way repeated measure GLM and Tukey test for each date. All statistical computations were done in SPSS 20.0.

## Results

### Overall temporal patterns in traits

**Figure 2:**
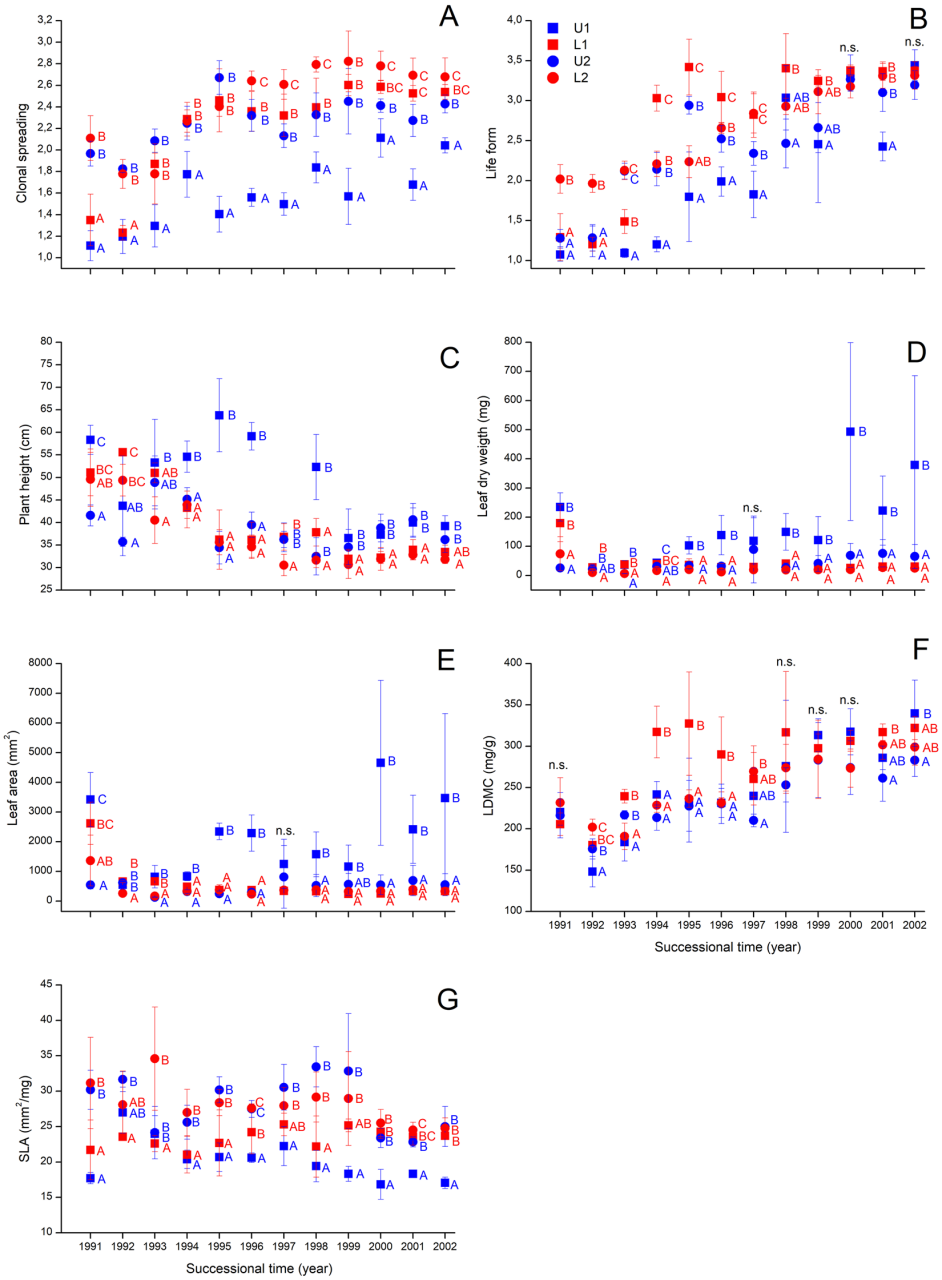
Temporal change of community-weighted means (CWMs) of vegetative traits. Notations: rectangle – sites at the eastern part of the reserve; circle – sites in the western part of the reserve; blue – sites laying near to the dune top; red – sites laying near to the dune slack. Significant differences were indicated by superscript letters (Univariate GLM and Tukey test). Clonal spreading ability was based on the four ordinal categories of the CLO-PLA database^29^; while life form was based on qualitative data for plant life form transformed to an ordinal scale based on the potential life span of each category.

We found marked temporal changes in the studied vegetative functional characteristics of the vegetation during the 12 years of secondary succession (Tables 1 and 2). We found a marked increase in community-weighted means (CWMs) of clonal spreading (Fig. 2A), life span (Fig. 2B), and LDMC (Fig. 2F). In parallel, we found a trend of decrease in the CWMs of plant height (Fig. 2C) and SLA (Fig. 2G). In the figures of leaf area (Fig. 2E) and leaf dry weight (Fig. 2D) the temporal pattern was not so obvious, but higher scores were typical, especially in U1 site in the later period of succession. In all cases we detected site specific effects and also the interaction of successional time and site was significant. The total cover showed high fluctuations during succession; the change in CWMs of LDMC, and clonal spreading were positively, while the CWM of plant height was negatively affected by the change of total cover (Table 1).

**Table 1:** Effects of site and successional time on community-weighted means (CWMs) of vegetative and regenerative traits in recovering sand grasslands.

Characteristic	Successional time		Site		Interaction		CTC
Community weighted mean (CWM)	*F*	*p*	*F*	*p*	*F*	*p*	*R*
***Vegetative traits***							
SLA	12.274	**	60.593	***	4.714	***	
LDMC	57.931	***	9.756	**	3.432	**	0.36^*^
Leaf area	28.405	***	21.077	***	9.988	***	
Leaf dry weight	20.668	**	12.904	***	3.388	**	
Clonal spreading	193.428	***	117.967	***	10.369	***	0.33^*^
Plant height	61.417	***	52.348	***	6.096	***	−0.46^**^
Life form	198.498	***	24.493	***	24.654	***	
***Regenerative traits***							
Flowering start	11.513	**	4.282	*	7.485	***	
Flowering period	16.859	**	16.141	***	7.599	***	−0.35^*^
Wind pollination	34.429	***	13.796	***	20.452	***	0.41^**^
Insect pollination	29.802	***	13.502	***	17.841	***	−0.42^**^
Self-pollination	26.906	***	19.505	***	6.583	***	
Seed weight	43.689	***	29.802	***	12.994	***	
Terminal velocity	24.842	***	26.460	***	11.401	***	−0.46^**^
Seed bank persistence	18.188	**	5.106	*	7.117	***	−0.44^**^

Two-way repeated measure GLM with ‘site’ as fixed and ‘successional time’ as repeated measure factor. Notations: CTC – correlation with total cover. Significance levels: n.s. = non-significant; ^*^0.01 ≤ p < 0.05; ^**^0.001 ≤ p < 0.01, ^***^p < 0.001.

**Figure 3:**
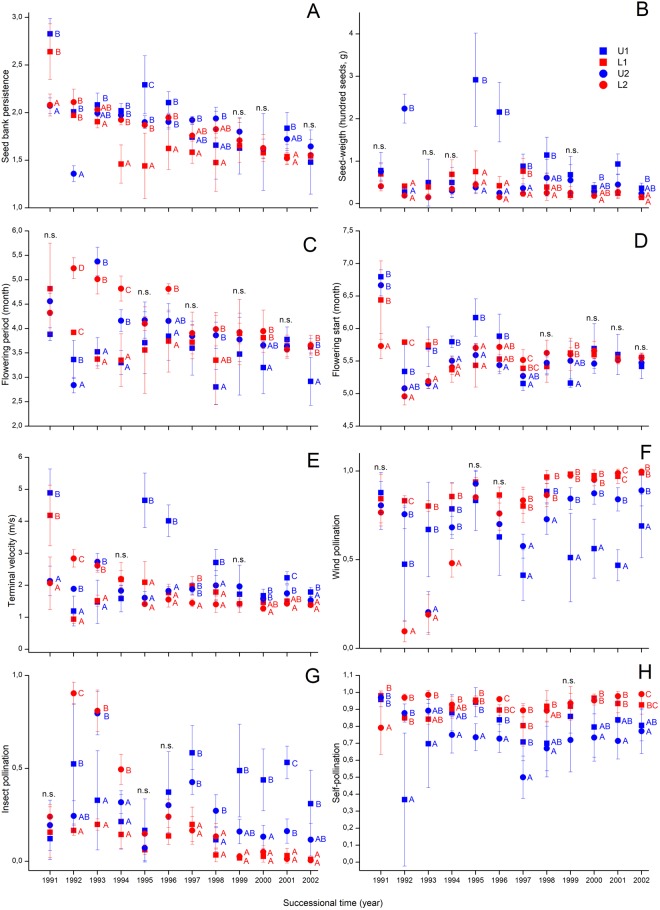
Temporal change of community-weighted means (CWMs) of regenerative traits. Notations: rectangle – sites at the eastern part of the reserve; circle – sites in the western part of the reserve; blue – sites laying near to the dune top; red – sites laying near to the dune slack. Significant differences were indicated by superscript letters (Univariate GLM and Tukey test). Seed bank persistence was based on the ordinal classification of Thompson *et al*.^33^ with three categories; while wind pollination, insect pollination and self-pollination values are the rate of presence/absence data from LEDA^25^ and BiolFlor^30^.

All of the CWMs of regenerative traits changed with successional time (Table 1). We found an increase in the CWMs of wind pollination (Fig. 3F), and a decrease in that of flowering start (Fig. 3D) and flowering period (Fig. 3C) regardless to site, but the latter two variables were characterised with a sharp drop in scores between the first and second year. In contrast, we found a temporal decrease in the CWMs of seed bank persistence (Fig. 3A) and terminal velocity (Fig. 3E). For the CWMs of seed weight (Fig. 3B) and insect pollination (Fig. 3G), the temporal trends were not obvious. For all studied traits, high fluctuations in scores were typical during the course of succession and a strong site-dependent effect and significant effect of the interaction between successional time and site were validated (Table 1). The total cover correlated positively with the CWM of wind pollination but negatively with the CWMs of flowering period, insect pollination, terminal velocity and seed bank persistence (Table 1).

**Table 2:** Effects of site and successional time on the functional diversity and single-trait functional divergence of recovering sand grasslands.

Characteristic	Successional time		Site		Interaction		CTC
	*F*	*p*	*F*	*p*	*F*	*p*	*R*
**Multi-trait indices**							
**Overall**							
Functional richness	1.098	n.s.	3.129	n.s.	0.859	n.s.	−0.31^*^
Functional evenness	2.870	n.s.	3.805	^*^	0.922	n.s.	
Functional divergence	4.158	^*^	16.719	^***^	3.756	^**^	
**For vegetative traits only**							
Functional richness	63.362	^***^	3.952	^*^	8.866	^***^	
Functional evenness	3.389	n.s.	4.035	^*^	1.407	n.s.	
Functional divergence	4.412	^*^	17.190	^***^	4,041	^**^	
**For regenerative traits only**							
Functional richness	22.982	^**^	10.195	^**^	2.312	^*^	
Functional evenness	4.931	^*^	17.467	^***^	1.230	n.s.	
Functional divergence	7.789	^*^	3.770	^*^	7.198	^***^	
**Single trait functional divergence (FDVar)**							
*Vegetative traits*							
SLA	5.924	^*^	20.154	^***^	2.677	^*^	
LDMC	12.838	^**^	16.969	^***^	5.827	^***^	
Leaf area	17.530	^**^	4.065	^*^	1.920	n.s.	
Leaf dry weight	22.147	^**^	1.619	n.s.	4.694	^***^	
Clonal spreading	37.409	^***^	5.091	^*^	9.081	^***^	−0.45^**^
Plant height	11.603	^**^	10.038	^**^	3.926	^**^	−0.44^**^
Life form	26.666	^***^	14.807	^***^	14.505	^***^	
*Regenerative traits*							
Flowering start	3.532	n.s.	0.897	n.s.	3.288	^**^	
Flowering period	202.180	^***^	17.225	^***^	15.631	^***^	0.42^**^
Seed weight	48.525	^***^	13.956	^***^	7.989	^***^	
Terminal velocity	12.744	^**^	5.474	^**^	3.905	^**^	−0.44^**^
Seed bank persistence	167.288	^***^	10.547	^***^	13.072	^***^	

Two-way repeated measure GLM with ‘site’ as fixed and ‘successional time’ as repeated measure factor. Notations: CTC – correlation with total cover (Spearman rank-correlation). Significance levels: n.s. = non-significant; ^*^0.01 ≤ p < 0.05; ^**^0.001 ≤ p < 0.01, ^***^p < 0.001. For the nominal traits (pollination types) single-trait functional divergence was not calculated.

### Effect of vertical position on functional characteristics

Almost all studied functional characteristics were affected by the site (Tables 1 and 2). From 1997–98 onwards, from the vegetative traits the CWMs of leaf area, leaf dry weight, and plant height (Fig. 2), and the FDVars of LDMC, leaf dry weight, and life form were higher at the high positioned sites (U1 and U2, Supplementary Figure S1). From the regenerative traits the CWMs of insect pollination, seed weight, and terminal velocity (Fig. 3), and the FDVars of flowering start and seed weight were higher at the high-positioned sites (U1 and U2; Supplementary Figure S2). In contrast, the CWMs of clonal spreading (Fig. 2A), wind pollination and self-pollination (Fig. 3F,H), and the FDVar of flowering period (Supplementary Figure S2J) were higher at the low-positioned sites (L1 and L2). For the other traits no such clear separation was detected along the vertical position and they were characterised by high year-to-year fluctuations.

### Patterns of vegetative and regenerative multi-trait functional diversity

**Figure 4:**
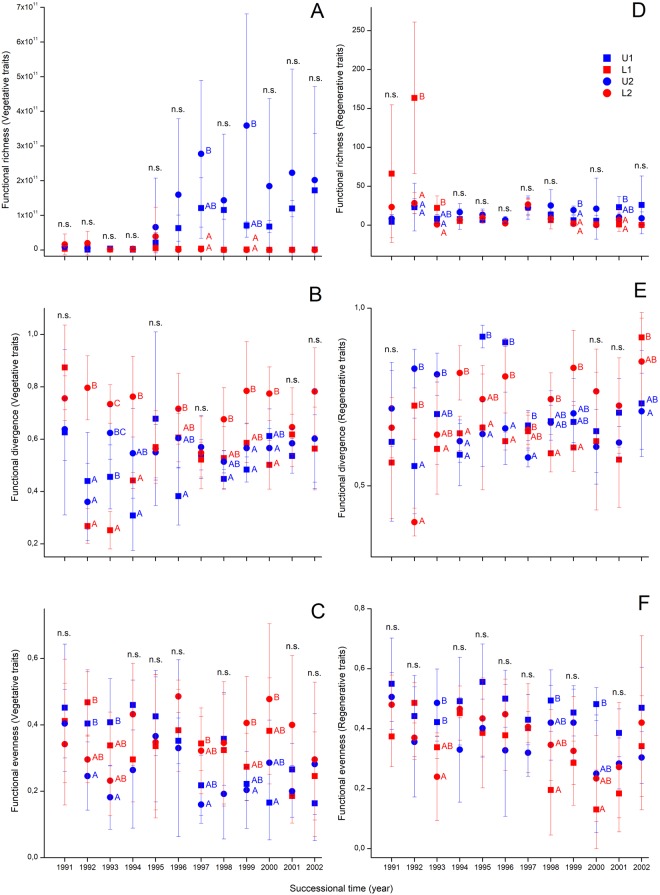
Temporal change of multi-trait indices of functional richness (FRich), functional divergence (FDiv) and functional evenness (FEve) for the vegetative (on the left) and regenerative traits (on the rigth). Notations: rectangle – sites at the eastern part of the reserve; circle – sites in the western part of the reserve; blue – sites laying near to the dune top; red – sites laying near to the dune slack. Significant differences were indicated by superscript letters (Univariate GLM and Tukey test).

Overall functional richness and evenness were not affected by successional time (Table 2). Overall functional divergence was significantly affected by successional time, site and their interaction, but it displayed quite large fluctuations especially in the first few years. The functional richness and evenness of vegetative and regenerative traits, when analysed separately, were mostly significantly affected by the successional time, site and their interaction (Table 2, Fig. 4). The functional evenness of vegetative traits was only affected by the site, while that of regenerative traits was affected both by successional time and site, but was not affected by their interaction and displayed a slightly decreasing trend with high fluctuations (Table 2). Except for the overall functional richness none of the multi-trait indices were correlated with the total cover (Table 2.).

In the early years, we found very low scores for functional richness of vegetative traits (Fig. 4A). After some years, scores for functional richness increased considerably on sites near to the dune top (U1, U2) compared to sites near to the dune slacks (L1, L2). For the functional richness of the regenerative traits, we found somewhat opposite trends, but in both cases a divergent vegetation development was also typical (Fig. 4D). For the functional divergence, there were some overall temporal increase in values with somewhat higher scores in lower lying sites (Fig. 4B,E). For functional evenness, a similar, but opposite, pattern was detected (Fig. 4C,F).

## Discussion

### Temporal patterns in traits

We hypothesised that the early vegetation development is characterised by stochastic processes displayed by high fluctuations of trait values, but later vegetation development is driven by the interaction of biotic and abiotic filtering displayed in a divergent vegetation development of low (L1 and L2) and high positioned (U1 and U2) sites. These assumptions were mostly confirmed by our results as especially in the first two to three years most traits displayed high fluctuations and no clear patterns for divergent vegetation development was found for most of the studied traits. In secondary succession, there are in general two sources of plant establishment – spatial dispersal of propagules and establishment from the local propagule banks (i.e. in most cases from local soil seed banks^39^). Following grazing by domestic goose, which also causes elevated soil fertility by phosphorous and potassium accumulation, the first few years are characterised by short-lived plant assemblages recruited both from the seed banks and spatial dispersal^35^. Populations of short-lived species are strongly influenced by the year-to-year fluctuations in weather (mostly by changes in precipitation) and are not able to form a stable plant community without regular propagule input and disturbance^40^. Thus, in the course of succession the short-lived weedy assemblages are replaced by more stress-tolerant, frequently clonally spreading perennials^41^. In terms of traits, this trend was also validated by our data. We found increasing rates of clonality and perenniality in the studied time period of secondary succession. This shift causes a decrease in the availability of free regeneration gaps and in the species turnover, and also slowed-down vegetation succession^42^.

### Traits and community assembly

We hypothesised that there will be different temporal patterns in the functional diversity of regenerative and vegetative traits. This assumption was only weakly supported by our results. While there were some distinct patterns found for the functional richness of vegetative and regenerative traits, for functional divergence and evenness no clear distinctive pattern was detected. However, it should be noted that an overall increase in functional divergence was detected both in vegetative and regenerative traits. Increasing functional divergence – especially for those traits which are strongly related to competition – is generally considered as a signal of increased niche differentiation and increased magnitude of competition^43,44^. It was stressed by Butterfield *et al*.^14^ that multi-trait indices may not be strong predictors of community change compared to single traits related to environmental gradients. This may be because multi-trait indices combine several single traits displaying contrasting temporal trends during succession, and it was also demonstrated by our results.

Similar temporal changes in life forms, seed bank persistence (i.e. seed longevity), LDMC and SLA were also validated by Purschke *et al*.^16^, but on a much longer time-scale in a space-for-time analysis. For LDMC and SLA the same trends were detected also by Kelemen *et al*.^23^. A decreasing trend in flowering period and flowering start was also detected by Douma *et al*.^17^. There were some traits like life-form (as a proxy of the life-span of plants), LDMC, SLA, seed bank persistence or flowering period, where strong increasing or decreasing temporal trends in their CWMs were detected, but we observed no divergent or convergent change. This may suggest that the changes in the CWMs of these traits may be rather general features of secondary succession.

In former research explaining community assembly two contrasting views are mentioned by Schleicher *et al*.^19^. First, the (trait-) neutral theory of species assembly explains the community assembly as a stochastic process (governed by priority vs. mass effects and stochastic population dynamics) which outcome is strongly influenced by the locally available species pool and site history in form of the legacy of former management and species composition (i.e. soil fertility and seed banks^39,45,46^). Second, the theory of functional filtering suggests that community assembly is trait-driven, which phenomenon is well expressed by the divergent or convergent vegetation development significantly differing from a random assembly^47^. In our study we found evidence for both theories. The vegetation of the first few years (especially the first year) was strongly influenced by the legacy of former vegetation and by local dispersal and establishment processes. For many traits we found that sites in similar topographic position (i.e. sites with different vertical position in eastern and western part of the national reserve) were more similar to each other than to sites with different topographic but similar vertical position. This is clearly visible for the CWMs of clonal spreading, leaf area, SLA, seed bank persistence, or terminal velocity. Thus, the first few years’ vegetation development can be at least partly explained by the trait neutral theory of vegetation development. In most cases this effect was diminished later on, or even an effect of filtering was detected in form of convergent change in some traits like clonal spreading, plant height, leaf area, terminal velocity, or pollination types in sites with similar vertical but different topographical position.

To sum up, we found that both trait neutrality and filtering effects can be tracked in the vegetation changes during the first period of secondary grassland succession. Initially, species establishment is influenced by local abiotic conditions and is a highly stochastic process that is reflected by the high fluctuations in trait values. Later, with increasing rate of clonality and perenniality, the community became more stable and the species assembly became governed by the interaction of abiotic and biotic filtering (i.e. altered competitive ability of species under different environmental stress) expressed clearly by divergent vegetation development (i.e. divergence in CWMs and FDVars of respective traits) of sites with different vertical position. High fluctuations in some trait values, however, were typical during the whole study period, which underlines that the effect of stochastic processes was especially important also in later stages in vegetation development.

## Electronic supplementary material

Supplementary information

## Data Availability

The authors intend to archive all data in Dryad Digital Repository upon acceptance.
