# Supplementary material for: Both trait-neutrality and filtering effects are validated by the vegetation patterns detected in the functional recovery of sand grasslands
Source: Sci Rep. 2018 Sep 12;8:13703. doi: 10.1038/s41598-018-32078-x (PMC6135751; doi:10.1038/s41598-018-32078-x)

Supplementary information

Both trait-neutrality and filtering effects are validated by the vegetation patterns detected in the functional recovery of sand grasslands

Török P., Matus G., Tóth E., Papp M., Kelemen A., Sonkoly J., Tóthmérész B.

Figure S1. Temporal change of single trait functional variance (FDvar) of vegetative and regenerative traits. Notations: rectangle – sites at the eastern part of the reserve, circle – sites in the western part of the reserve. blue – sites laying near to the dune top, red – sites laying near to the dune slack. Significant differences were indicated by superscripted letters (Univariate GLM and Tukey test).

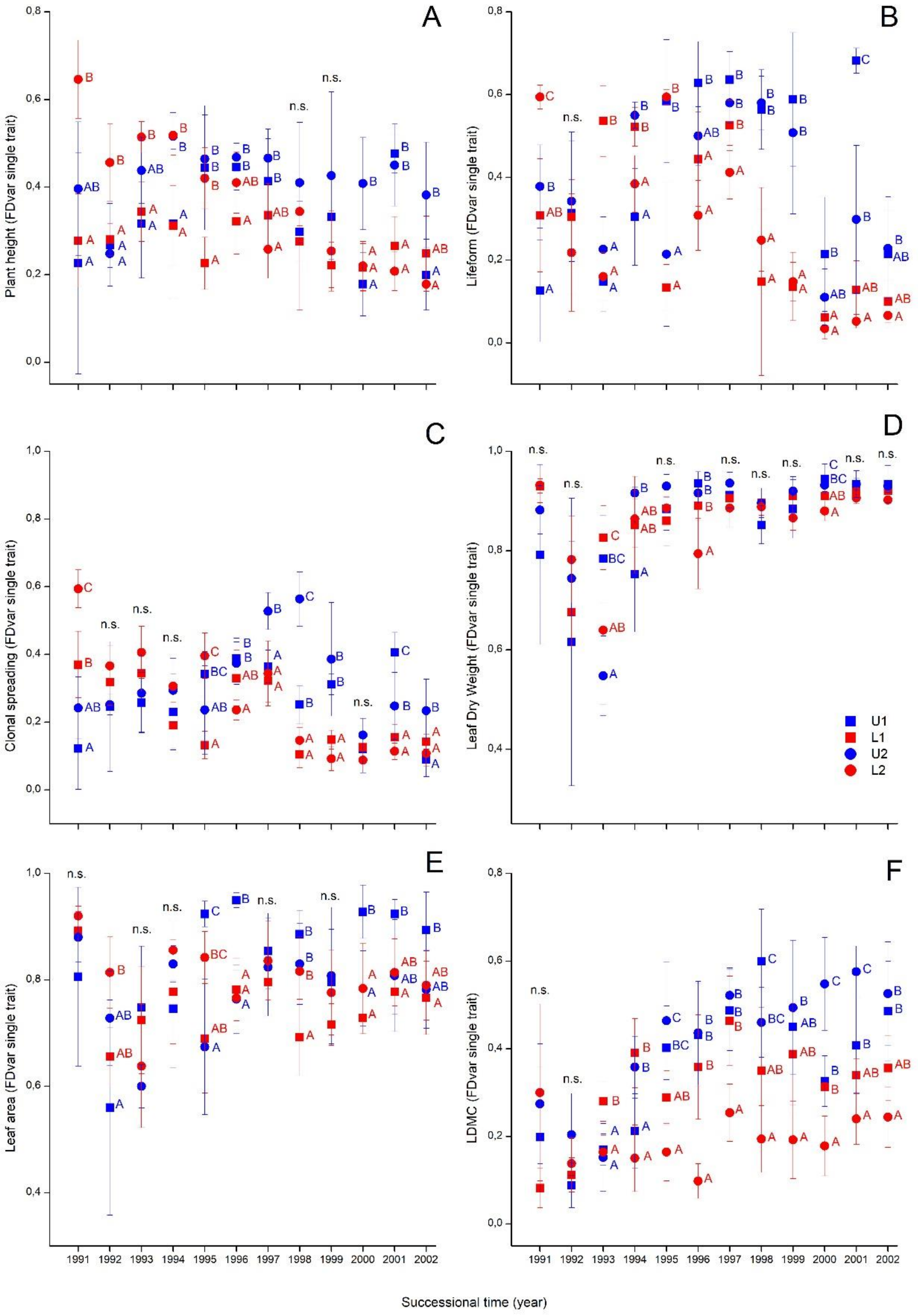

Figure S2. Temporal change of single trait functional variance (FDvar) of vegetative and regenerative traits. Notations: rectangle – sites at the eastern part of the reserve, circle – sites in the western part of the reserve. blue – sites laying near to the dune top, red – sites laying near to the dune slack. Significant differences were indicated by superscripted letters (Univariate GLM and Tukey test).

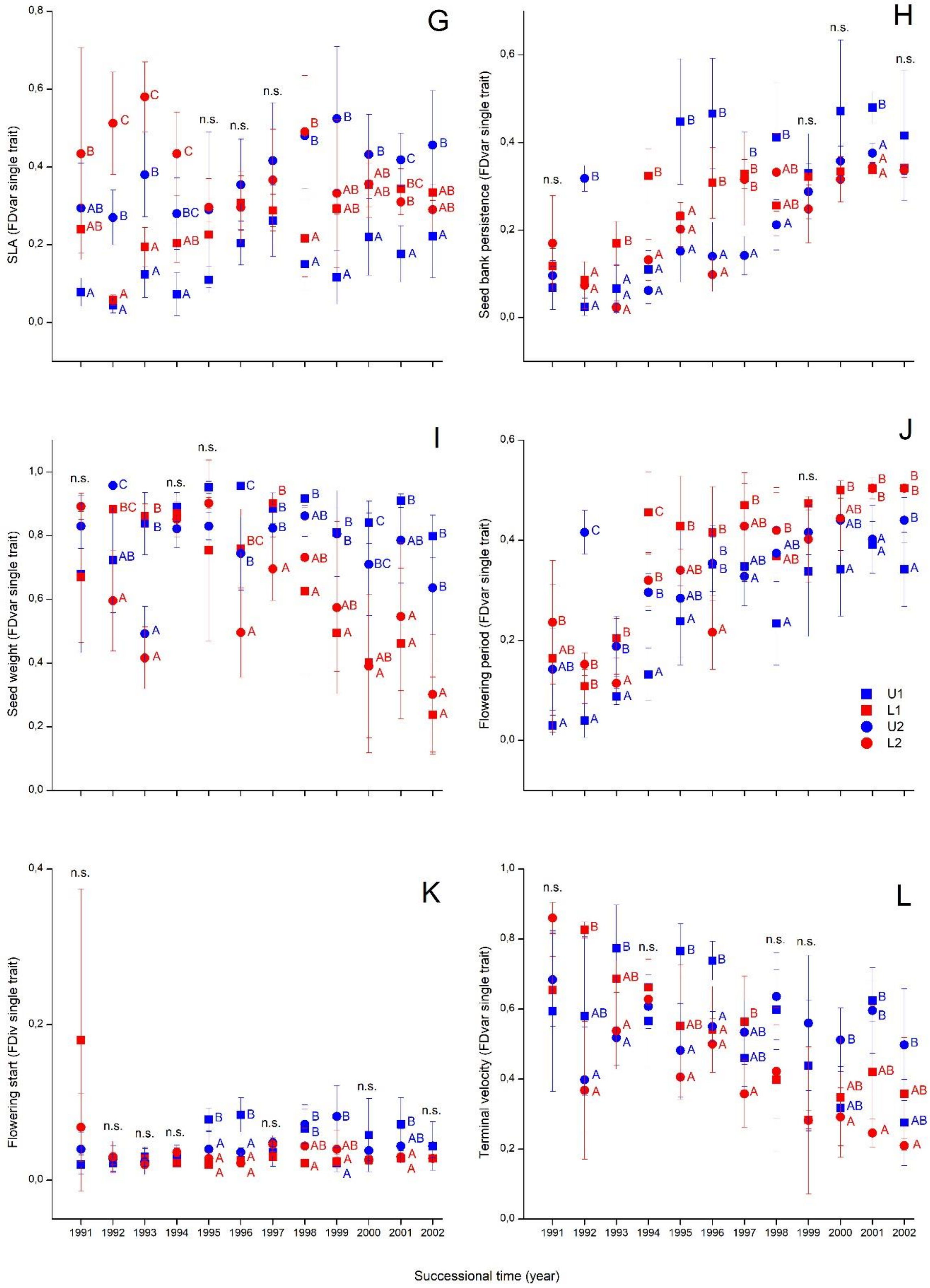

Supplement: Supplementary file 1 — Supplementary information [file 41598_2018_32078_MOESM1_ESM.pdf]
